# Genetic Continuity in the Franco-Cantabrian Region: New Clues from Autochthonous Mitogenomes

**DOI:** 10.1371/journal.pone.0032851

**Published:** 2012-03-19

**Authors:** Alberto Gómez-Carballa, Anna Olivieri, Doron M. Behar, Alessandro Achilli, Antonio Torroni, Antonio Salas

**Affiliations:** 1 Unidade de Xenética, Departamento de Anatomía Patolóxica e Ciencias Forenses, Instituto de Medicina Legal, Facultade de Medicina, Universidade de Santiago de Compostela, Galicia, Spain; 2 Dipartimento di Biologia e Biotecnologie “L. Spallanzani,” Università di Pavia, Pavia, Italy; 3 Rambam Health Care Campus, Haifa, Israel; 4 Dipartimento di Biologia Cellulare e Ambientale, Università di Perugia, Perugia, Italy; University of Florence, Italy

## Abstract

**Background:**

The Late Glacial Maximum (LGM), ∼20 thousand years ago (kya), is thought to have forced the people inhabiting vast areas of northern and central Europe to retreat to southern regions characterized by milder climatic conditions. Archaeological records indicate that Franco-Cantabria might have been the major source for the re-peopling of Europe at the beginning of the Holocene (11.5 kya). However, genetic evidence is still scarce and has been the focus of an intense debate.

**Methods/Principal Findings:**

Based on a survey of more than 345,000 partial control region sequences and the analysis of 53 mitochondrial DNA (mtDNA) genomes, we identified an mtDNA lineage, HV4a1a, which most likely arose in the Franco-Cantabrian area about 5.4 kya and remained confined to northern Iberia.

**Conclusions/Significance:**

The HV4a1a lineage and several of its younger branches reveal for the first time genetic continuity in this region and long-term episodes of isolation. This, in turn, could at least in part explain the unique linguistic and cultural features of the Basque region.

## Introduction

In the last decade, numerous studies have evaluated the distribution and extent of variation of mitochondrial DNA (mtDNA) in European populations, and evidence of the overwhelming importance of the Franco-Cantabrian refugium for the re-peopling of much of Western and Northern Europe at the beginning of the Holocene has been obtained by the age estimates and geographic distributions of certain mtDNA haplogroups.

In 1998, an early study proposed that haplogroup V was an autochthonous European haplogroup that originated in northern Iberia or southwestern France at about the time of the Younger Dryas [1]. The follow-up study of Achilli et al. [2] investigated numerous sub-branches of haplogroup H and demonstrated that haplogroups H1 and H3 were characterized by (i) frequency peaks centered in Iberia and surrounding areas and (ii) higher diversity values for this region than in any other area in Europe. As suggested by their phylogeographic distributions and coalescence ages, these haplogroups most likely contributed to the late-glacial expansion of hunter-gatherers who repopulated Central and Northern Europe about 11–13 kya after the Late Glacial Maximum (LGM). Pereira et al. [3] targeted the coding region of individuals carrying haplogroup H, sampled from Europe, the Caucasus and the Near East, identifying sub-clades also signaling a population re-expansion from the Iberian refugium. Álvarez-Iglesias et al. [4] provided new evidence in support of the important role played by the Franco-Cantabrian refuge area corroborating previous findings. In addition, the same authors identified a minor autochthonous haplogroup from the Basque Country with a coalescence age of ∼1.3 kya, indicating recent isolation events in the region. Some recent articles have however challenged these findings, and therefore, the role of the Franco Cantabrian region as one of the most important European refuge area after the LGM still remains under debate [5], [6].

Recently, a few (mostly control region) mtDNA sequences belonging to haplogroup HV4 were reported in the literature and databases (mainly GenBank) [2], [4], [6]–[8]. However, these studies did not perceive that a minor sub-clade of HV4 might have been more prevalent in the Basque Country and surrounding areas (Cantabria, southwestern France), coinciding with the Franco-Cantabrian area.

In this study, to shed some light on the origin of HV4 and its derivatives, we surveyed its distribution in a wide range of European (and neighboring) populations and evaluated its sequence diversity by analyzing 53 HV4 mitochondrial genomes.

## Methods

### Ethics statement

Written informed consent was obtained from all sample donors. Analysis of mtDNA sequences was approved by the three different institutional review boards involved in sampling recruitment: the Ethical committee of the University of Santiago de Compostela, the Ethics Committee for Clinical Experimentation of the University of Pavia (Board minutes of October 5, 2010), and the Institutional Review Boards of the University of Pennsylvania and the United States Department of Health and Human Services. Moreover, the study conforms to the Spanish Law for Biomedical Research (Law 14/2007- 3 of July).

### Analysis of sequence variation and data-mining

**Figure 1:**
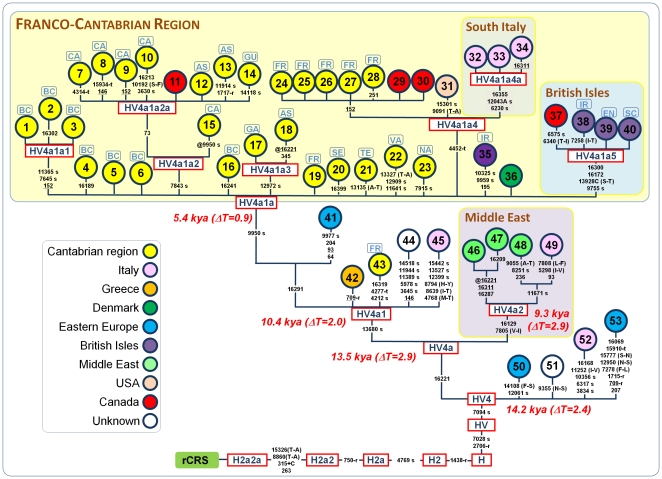
Maximum parsimony tree of entire mtDNA genomes belonging to haplogroup HV4. The mutations are displayed along the branches. The position of the revised Cambridge reference sequence (rCRS) is indicated for reading off sequence motifs [15]. All mutations are transitions unless a suffix specifies a transversion (A, C, G, T), an insertion (+), a synonymous substitution (s), a mutational change in tRNA (-t), a mutational change in rRNA (-r), a non-coding variant located in the mtDNA coding region (-nc) or an amino acid replacement (indicated in round brackets). Recurrent mutations within the phylogeny are underlined. The prefix “@” indicates a back mutation. Mutational hotspot variants such as 16182, 16183, or 16519, or a variation around position 310 and length or point heteroplasmies were not considered for the phylogenetic reconstruction. Divergence times correspond to the ML estimates reported in Table 1. Population codes (blue squares on top of circles) for the Cantabrian region: BC = Basque Country, CA = Cantabria, AS = Asturias, GA = Galicia, TE = Teruel, VA = Valladolid, SE = Segovia, GU = Guadalajara; FR = France; NA = Navarra.

More than 134,800 control region profiles were compiled from the literature (with a special focus on Europe, >27,000 sequences; see the references in the Supplemental Data) and searched for the putative diagnostic HV4a1a sequence motif C16221T- C16291T. Only 21 HV4a1a sequences were observed in this database, but most of them (∼62%) were located in the Franco-Cantabrian area, and the remaining mainly among French Canadians and in the USA. In addition, more than 8,700 mitochondrial genomes were recruited from the literature and GenBank [9], but only 14 were found to belong to HV4; of these, four, all from the Franco-Cantabrian area, were allocated to the sub-clade HV4a1a (then sharing C16221T and T9950C on top of C16291T). Given the low mutation rate at nucleotide position 16221 (one single hit in the list of allele-specific mutation rates of Soares et al. [10]), the sequence motif for HV4a1a can be considered to be very robust. Therefore, searching for this sequence motif provides a unique opportunity to survey HV4a1a candidates in local databases. By searching our local DNA databanks, which include 211,156 DNAs, for sequences sharing the control-region mutations C16221T and/or C16291T, we detected 55 additional control-region sequences, and 39 of these were completely sequenced in the present study (plus three additional ones that did not contribute to the HV4a1a branch; Figure 1 and Table S1). DNA sequencing was carried out as described in Cerezo et al. [8]. Mitochondrial DNA genomes were submitted to GenBank (accession numbers: JN214391 to JN214429.

### Statistical analysis and molecular dating

The spatial geographical representation of haplogroup HV4a1a was obtained using Surfer 8.0 software (http://www.goldensoftware.com). We followed the commonly used inverse-squared distance method for interpolating frequency values; other interpolated methods yielded virtually the same results. The haplogroup frequencies are presented in a regular grid covering Europe.

**Figure 2:**
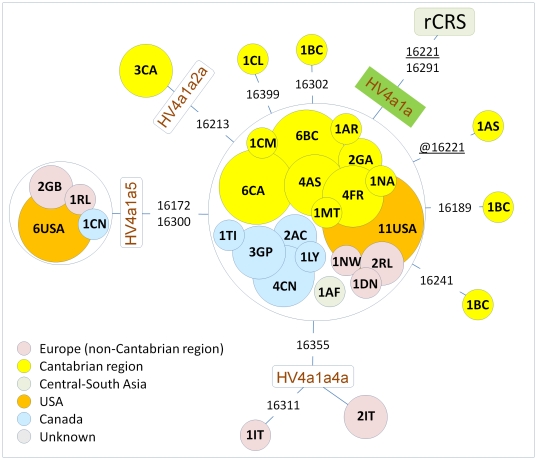
Maximum parsimony tree of available HV4a1a control region sequences (HVS-I sequence motif: C16221T-C16291T). This tree includes the ones inferred from the complete genomes considered in Figure 1. The area of the circles is proportional to the sample size. Population codes (within circles) are as follows: Iberia: CA = Cantabria (central-northern), CL = Castile León (north-central), BC = Basque Country (central-northern), CM = Castile La Mancha (north-central), AR = Aragón (north-central, GA = Galicia (north-western), AS = Asturias (north-central), NA = Navarra (north-central), France: FR = France, MT = Martinique; Canada: CN = Canada, GP = Gaspesia (south-east), AC = Acadia (south-east), LY = Loyalists (south-east), TI = Prince Edward Island, Tignish (south-east), IT = Italy; GB = Great Britain, RL = Ireland, USA = United States of America, NW = Norway; DN = Denmark; AF = Afghanistan.

Maximum parsimony trees were built for complete HV4 genomes (Figure 1) and HVS-I segments (Figure 2). The time to the most recent common ancestor (TMRCA) for each cluster was calculated by computing the averaged distance (ρ) of all the haplotypes in a clade to the respective root haplotype. Heuristic estimates of the standard error (σ) was calculated from an estimate of the genealogy [11]. Calculations were obtained using the entire mtDNA genomes but excluding hotspot mutations such as 16182C, 16183C and 16519. Mutational distances were converted into years using the corrected evolutionary rate proposed by Soares et al. [10]. We also compared ρestimates with those obtained using the maximum likelihood (ML) approach. PAML 4.4 software was employed for ML estimates assuming the HKY85 mutation model (ignoring indels) with gamma-distributed rates (approximated by a discrete distribution with 32 categories) (http://abacus.gene.ucl.ac.uk/software/paml.html, [12]). Age estimates were again extrapolated using the corrected mutation rate of Soares et al. [10]. Standard deviations of age estimates are noted as ΔT along the text. As observed in Table 1, both methods for age estimation yielded very similar results (their ratio is at least 0.74); in the text, for the sake of clarity, we refer to the values obtained from ρ estimates.

**Table 1:** Molecular divergences and age estimates (Maximum Likelihood and ρ Statistics) for HV4 and its sub-clades.

Haplogroup	n^a^	ML	S.E.	T (Ky)^b^	ΔT (Ky)^b^	ρ	σ	T (Ky)^b^	ΔT (Ky)^b^
HV4	53	5.3	0.9	14.2	2.4	6.4	1.6	17.2	9.1
Hv4a	49	5.0	1.1	13.5	2.9	5.3	1.5	14.3	8.1
Hv4a1	45	3.9	0.7	10.4	2.0	4.3	1.3	11.5	6.8
Hv4a1a	38	2.1	0.3	5.4	0.9	2.6	0.5	6.8	2.8
Hv4a1a2	9	1.7	0.3	4.4	0.9	2.0	1.0	5.2	4.9
Hv4a1a2a	8	1.4	0.3	3.6	0.8	1.1	0.4	2.9	1.9
Hv4a1a3	2	1.0	0.7	2.7	1.9	-	-	-	-
Hv4a1a4	11	1.6	0.4	4.2	1.1	1.7	0.8	4.5	4.1
Hv4a1a4a	3	0.1	0.1	0.4	0.4	-	-	-	-
Hv4a1a5	4	0.5	0.3	1.4	0.8	0.8	0.4	1.9	2.2
Hv4a2	4	3.5	1.1	9.3	2.9	3.8	1.2	9.9	6.4

Delta T indicates the standard deviation computed using the two different age estimation methods.

a number of complete mtDNA sequences.

b using the corrected molecular clock proposed by Soares et al. (2009) for complete mtDNA genomes which is 0.0074±0.00019 substitutions per site.

## Results

### The phylogeny of HV4 mitochondrial genomes

To define the origin and relationships of haplogroup HV4, a total of 39 mtDNAs belonging to HV4 were completely sequenced and, together with 14 previously published HV4 sequences (Table S1), were incorporated in the tree illustrated in Figure 1. Overall the phylogeny of HV4 suggests an origin in Eastern Europe about 14.2 kya (ΔT = 2.4). The first split of HV4 distinguishes a main sub-clade (HV4a) defined by the variant C16221T, which in turn shows two main sub-haplogroups, referred here as to HV4a1 and HV4a2 (Figure 1). Sub-haplogroup HV4a2 is characterized by the sequence motif G7805A-G16129A; its geographic distribution suggests an origin somewhere in the Middle East about 9.3 kya (ΔT = 2.9). Some diffusions in the Mediterranean Basin cannot be disregarded as testified by an Italian representative sequenced in the present study (#49 in Figure 1).

The main HV4a1 sub-branch, namely HV4a1a, shows a different geographic distribution and encompasses the large majority of the HV4 genomes (∼75%); most of these genomes were from subjects who lived along the Cantabrian Sea coastline, with frequencies peaks in the Basque Country and in the Department of Cantabria (an area that is bordering with the West frontier of the Basque Country). The coalescence age of HV4a1a was estimated to be 5.4 kya (ΔT = 2.0). As inferred from the phylogeny (Figure 1), HV4a1a is more diverse in the Franco-Cantabrian area than in any other place. As reported in Table 1, there are at least three sub-clades (HV4a1a1, HV4a1a2 and HV4a1a3) that arise in this area in the period ranging from 2.7 (ΔT = 1.9) to 4.4 kya (ΔT = 0.9). There is also a young sub-clade, HV4a1a4a, which has a local distribution in South Italy with a coalescence age of 0.4 kya (ΔT = 0.4), and another minor clade in the British Isles, HV4a1a5, with a TMRCA of 1.4 kya (ΔT = 0.8). The major influence that Spaniards had in southern Italy, particularly in Naples and the surrounding areas, could easily explain the mtDNA connection observed with the Franco-Cantabrian area, while the presence of HV4a1 in the British Isles (and North America), indicates an older predominant expansion of this clade along the Atlantic façade.

### Partial control region data

**Figure 3:**
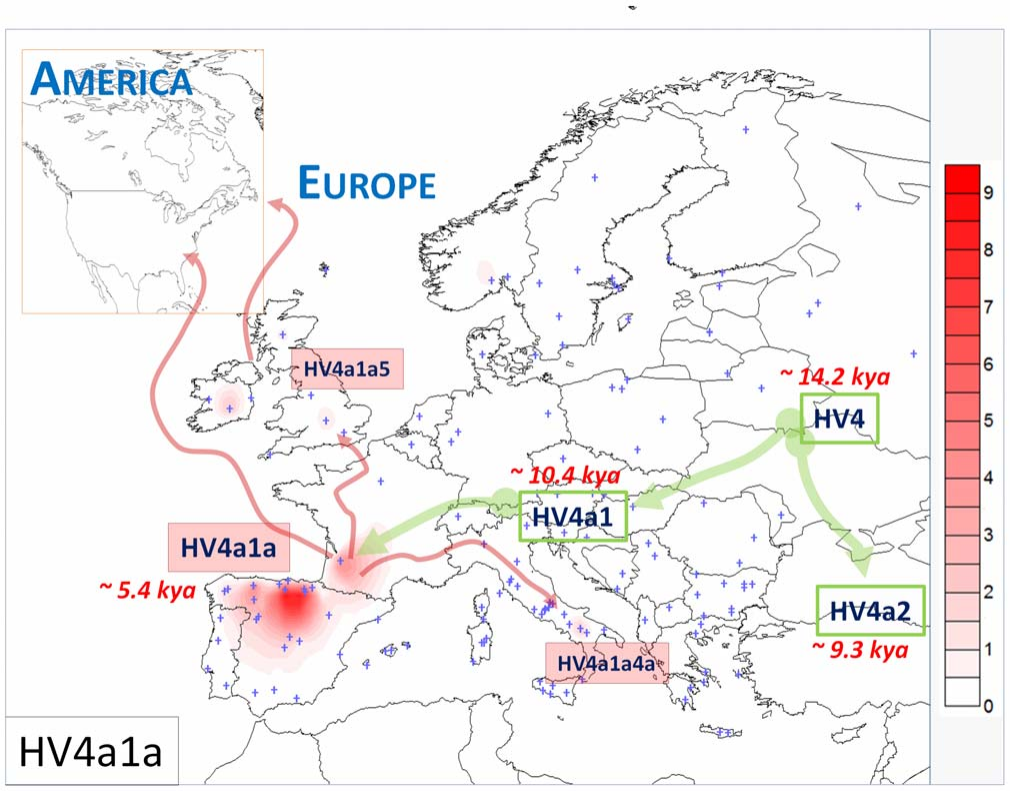
Map of Europe showing the frequency distribution of haplogroup HV4a1a. Blue crosses represent the location of the sampling points (see Table S1 for more information). Arrows represent a tentative reconstruction of the pre-historical and historical movements of HV4 and its sub-lineages across Europe and America. The scale indicates the absolute frequency of the HV4a1a mtDNAs in the regions sampled.

Only 76 control-region mtDNA profiles were found in a large worldwide database (including the ones analyzed for the new complete genomes). The phylogeny of all available control-region segments is represented in Figure 2. Surprisingly, 35 of these (∼46%) originated in the Cantabrian cornice, from the westernmost edge of Iberia (Galicia, eastwards to Asturias, Cantabria, the Basque Country and southwestern France), as also attested by the highest peak in the frequency map of Figure 3. Interestingly, in addition to the Cantabrian lineages, a total of 12 mtDNAs (∼16%) were observed in a sample from east Canada [13]. Two of these French Canadian mtDNAs were found in the Acadians, a group of people who settled in eastern Canada in the 1700s. Historical documentation indicates that a number of Acadians were probably farmers originally from western France. Given their historical French connection, the three other Canadian mtDNAs (two Gaspesians and one Loyalist) were most likely of French origin. Benjamin Sulte, in the “Origin of the French Canadians” (1906, p. 99), says that the Acadian dialect indicated their place of origin as being around the Bay of Biscay and at the mouth of the Loire River. Geneviève Massignon (in *Les Parlers Francais D'Acadie*) suggests that they came from the Loudunais area in northeastern Poitou (northern section of today's Vienne department) (taken from: http://www.acadian-cajun.com/colorig.htm). Therefore, the Canadian HV4a1a haplotypes, most likely of French origin, indicate a direct connection between the Basque Country and western France. The basal motifs of HV4a1a and the HV4a1a5 clade were also found in the USA (23%), but as the ones observed in Canada, they did not show signs of substantial internal diversity indicating that only a few (even only two) founders might have crossed the Atlantic in Colonial times (see also Figure 1).

## Discussion

The post-glacial debate and the odd distribution of HV4 mtDNAs in worldwide databases inspired this study under the hypothesis that a minor sub-clade of HV4, here referred to as HV4a1a, might have witnessed events of (pre)historical isolation in the Franco-Cantabrian region, thus perhaps explaining the uniqueness of some cultural features of the Basque Country.

A tentative reconstruction of the pre-historical and historical movements of HV4 and its sub-lineages across Europe and America is summarized in Figure 3, where the geographic distribution of HV4a1a also indicates the existence of a demographic episode of isolation in the Franco-Cantabrian region from about 5.4 kya (ΔT = 0.9). The presence of different sub-clades of HV4a1a in the region demonstrates genetic continuity in this region, probably up to very recent times. The present study demonstrates once more that focusing on specific mtDNA lineages can help to unravel past demographic events in human populations; events that might pass by completely unnoticed if large-scale genomic approaches are used [14].

## Supporting Information

Table S1 **Mitochondrial DNA data generated in the present study.** References and other data resources used in the present study are also given.
(XLS)
